# FLASH Radiotherapy for the Treatment of Symptomatic Bone Metastases (FAST-01): Protocol for the First Prospective Feasibility Study

**DOI:** 10.2196/41812

**Published:** 2023-01-05

**Authors:** Emily C Daugherty, Anthony Mascia, Yong Zhang, Eunsin Lee, Zhiyan Xiao, Mathieu Sertorio, Jennifer Woo, Claire McCann, Kenneth Russell, Lisa Levine, Ricky Sharma, Deepak Khuntia, Jeffrey Bradley, Charles B Simone II, John Perentesis, John Breneman

**Affiliations:** 1 Department of Radiation Oncology University of Cincinnati Cincinnati, OH United States; 2 Cancer and Blood Disease Institute Cincinnati Children's Hospital Cincinnati, OH United States; 3 Varian, A Siemens Healthineers Company Palo Alto, CA United States; 4 Department of Radiation Oncology Emory University School of Medicine Atlanta, GA United States; 5 Department of Radiation Oncology New York Proton Center New York, NY United States

**Keywords:** bone metastases, FLASH, proton therapy, external beam radiotherapy, palliative radiotherapy, extremities, pain relief, ultra-high dose rate, radiation therapy, cancer treatment, toxicity, oncology, radiotherapy

## Abstract

**Background:**

In preclinical studies, FLASH therapy, in which radiation delivered at ultrahigh dose rates of ≥40 Gy per second, has been shown to cause less injury to normal tissues than radiotherapy delivered at conventional dose rates. This paper describes the protocol for the first-in-human clinical investigation of proton FLASH therapy.

**Objective:**

FAST-01 is a prospective, single-center trial designed to assess the workflow feasibility, toxicity, and efficacy of FLASH therapy for the treatment of painful bone metastases in the extremities.

**Methods:**

Following informed consent, 10 subjects aged ≥18 years with up to 3 painful bone metastases in the extremities (excluding the feet, hands, and wrists) will be enrolled. A treatment field selected from a predefined library of plans with fixed field sizes (from 7.5 cm × 7.5 cm up to 7.5 cm × 20 cm) will be used for treatment. Subjects will receive 8 Gy of radiation in a single fraction—a well-established palliative regimen evaluated in prior investigations using conventional dose rate photon radiotherapy. A FLASH-enabled Varian ProBeam proton therapy unit will be used to deliver treatment to the target volume at a dose rate of ≥40 Gy per second, using the plateau (transmission) portion of the proton beam. After treatment, subjects will be assessed for pain response as well as any adverse effects of FLASH radiation. The primary end points include assessing the workflow feasibility and toxicity of FLASH treatment. The secondary end point is pain response at the treated site(s), as measured by patient-reported pain scores, the use of pain medication, and any flare in bone pain after treatment. The results will be compared to those reported historically for conventional dose rate photon radiotherapy, using the same radiation dose and fractionation.

**Results:**

FAST-01 opened to enrollment on November 3, 2020. Initial results are expected to be published in 2022.

**Conclusions:**

The results of this investigation will contribute to further developing and optimizing the FLASH-enabled ProBeam proton therapy system workflow. The pain response and toxicity data acquired in our study will provide a greater understanding of FLASH treatment effects on tumor responses and normal tissue toxicities, and they will inform future FLASH trial designs.

**Trial Registration:**

: ClinicalTrials.gov NCT04592887; http://clinicaltrials.gov/ct2/show/NCT04592887

**International Registered Report Identifier (IRRID):**

DERR1-10.2196/41812

## Introduction

### Background

FLASH is an emerging radiation treatment modality that has shown promise for improving the therapeutic ratio by potentially reducing normal tissue toxicity when compared to conventional radiotherapy. FLASH treatment is delivered at ultrahigh dose rates of at least 40 Gy per second, which is approximately 1000 times the dose rate of conventional radiotherapy. In our study—the first clinical trial of FLASH—10 subjects will undergo FLASH treatment for painful bone metastases in the extremities, using a proton radiotherapy system.

The bone, liver, and lungs are the most common sites of metastases from cancer [1]. The pain caused by bone metastases can be severe and debilitating and compromises the quality of life of patients [2,3]. A multimodal approach is often required to adequately manage bone metastases [3], and this does not always adequately control pain.

Subjects with painful bone metastases benefit from radiation therapy [3-8]. Radiotherapy palliation for bone metastasis pain can reduce the use of analgesics [9], enhance mobility, and improve quality of life [10-12]. For painful bone metastases, a standard-of-care radiotherapy regimen is 8 Gy of radiation delivered in 1 fraction (a single treatment) via a medical linear accelerator [4]. Because of the toxicities inherent with ionizing radiation, achieving a favorable therapeutic ratio (balancing radiation-induced damage to the tumor while sparing normal tissue) is a key goal of radiotherapy prescriptions and planning for both radical treatment and palliative treatment.

### Study Intervention

Multiple preclinical models have shown that FLASH therapy, when compared to conventional radiotherapy, results in the increased protection of healthy tissue and cells and has equal or greater tumor cell kill rates [13-17]. Research is ongoing to understand the mechanisms underlying the observed benefits of FLASH. Data suggest that the lower levels of toxic oxygen reactive species in normal tissues may explain why less side effects may be produced by FLASH than by conventional radiotherapy [18]. The normal tissue–sparing effects of FLASH have been observed in several tissues and animal models, including mouse intestines [19], mouse skin [20], mouse lungs [21], mouse brains [18], and cat and pig skin [22].

Irradiating mouse lungs in vivo, Favaudon et al [21] found that a higher FLASH dose was required to induce radiation pneumonitis and fibrosis than the dose required for conventional radiotherapy (30 Gy for FLASH vs 17 Gy for conventional radiotherapy). Furthermore, tumor control was more easily achieved with FLASH. The tumor control rates were 70% at 27 Gy in FLASH and 20% at 15 Gy in conventional radiotherapy. At 27 Gy in FLASH, no pneumonitis was observed, whereas significant pneumonitis was observed at 15 Gy in conventional radiotherapy [21]. Similarly, Montay-Gruel et al [18] treated mouse brains in vivo. Subsequent follow-ups revealed that neurocognitive impairment developed in the 10-Gy conventional radiotherapy group but not in the 10-Gy FLASH group. Similarly, Cunningham et al [23] demonstrated that FLASH proton pencil beam scanning irradiation minimized radiation-induced leg contracture and skin toxicity in mice.

To our knowledge, there is only 1 peer-reviewed report [24] on the use of FLASH in humans—a case report of a single subject with cutaneous T-cell lymphoma who underwent extensive prior skin radiotherapy. This individual was treated with a single 15-Gy dose of FLASH, using electrons for a recurrent cutaneous lymphoma lesion. The lesion had a complete response to treatment, with minimal toxicity to the surrounding skin.

A standard of care for the radiotherapy palliation of painful bone metastases is 8 Gy of photon radiation delivered by a medical linear accelerator. Although there is no commercially available medical linear accelerator capable of delivering FLASH dose rates, some cyclotrons, which generate proton radiation for medical treatments, are capable of FLASH dose rates when operated in research mode. Our study will be carried out in a proton treatment facility delivering FLASH dose rate treatment to subjects with painful bone metastases in the extremities.

### Rationale and Risk Benefit

The implementation data acquired in this first-in-human investigation will contribute to developing the clinical workflow for FLASH and making this technology routinely available to radiation oncologists and their patients.

The prescription dose and fractionation in this study are the same as those in the standard of care for the palliation of bone metastases—8 Gy of radiation delivered in a single treatment [4]. A single radiotherapy fraction of 8 Gy was also used to administer conventional radiotherapy in prior clinical studies [25,26]. Based on the early data indicating that FLASH may be capable of controlling tumors as effectively as conventional radiotherapy [25,26], it is expected that the subjects with painful bone metastases treated using this study protocol will receive the same pain control benefit as that received by patients treated with conventional radiotherapy.

The subjects’ experience of treatment and the follow-up schedule were designed to be very similar to those of standard-of-care conventional radiotherapy. Some of the study activities will occur during regular visits that the subjects will have as part of their oncologic care. Thus, the time and social burden imposed by study participation will be minimal. FLASH will only be delivered to targets in the extremities, which are distant from visceral organs at higher risk of radiation toxicity. In this study, normal tissue toxicity due to FLASH is expected to be no more than (and potentially less than [21]) that historically observed with conventional radiotherapy. Toxicities of conventional radiotherapy have been evaluated in a prior benchmark multi-institutional prospective randomized clinical trial [9], in which a cohort of 455 patients (433 and 354 patients analyzed for acute and late toxicities, respectively) was treated with 8-Gy, single-fraction conventional radiotherapy for the palliation of painful bone metastases. Toxicities were scored using the Radiation Therapy Oncology Group (RTOG) acute and late morbidity Criteria [27]. Late toxicity (occurring >90 days after radiotherapy) was “rare (4%),” and no patients in the cited study had any grade 4 to 5 acute or late toxicities. The most common toxicity was gastrointestinal. Gastrointestinal toxicity should not be relevant to our feasibility study because radiotherapy will be delivered to the extremities, and no portion of the gastrointestinal tract will be in the radiation field. The acute toxicity that is most likely to occur in this study is radiation dermatitis within the irradiated field. In the prior cited study, of the 433 patients, there were 15 (3%) grade 1, 1 (<1%) grade 2, and no grade 3 acute skin toxicities. Hematologic and “other” (not specified in the prior studies) acute toxicities also occurred in the cited trial.

## Methods

### Trial Design

This clinical trial is a first-in-human feasibility study of proton FLASH (trial registration number: NCT04592887). Up to 10 adult subjects will undergo FLASH on a proton therapy system operated in research mode. Each subject will undergo FLASH treatment for up to 3 painful bone metastasis sites in the extremities. The feasibility of the clinical workflow will be evaluated. A combination of physician-reported and patient-reported outcomes will be used to assess toxicities and pain relief at scheduled time points throughout the study for the duration of the study participants’ lifetime or until they are lost to follow-up.

### Objectives

The primary objectives are to assess the workflow feasibility of FLASH therapy in a clinical setting and the toxicities of treatment. The secondary objective is to assess pain relief at the treated site(s).

### Setting

This clinical trial will be conducted at the Cincinnati Children’s/University of Cincinnati Health Proton Therapy Center—an academic hospital in Cincinnati, Ohio.

### Oversight and Compliance

The protocol was reviewed and approved by the US Food and Drug Administration (FDA) prior to participation by any subjects. This research will be conducted on a modified proton therapy device under an investigational device exemption (IDE) approved by the FDA.

### Ethics Approval

The FAST-01 protocol was reviewed and approved by the Cincinnati Children’s Hospital Institutional Review Board (reference ID: 2020-0030) prior to participation by any subjects. 

### Recruitment

Patients presenting for palliative radiotherapy to bone metastases will be screened for potential inclusion. In addition, copies of a flier (Multimedia Appendix 1) inviting patients to inquire about the study will be posted.

The radiation oncologists and their staff involved in the study will administer the informed consent procedures for the patients.

### Enrollment and Replacement

Enrollment for the study is expected to take 12 months.

All inclusion criteria must be met, and none of the exclusion criteria may be present for a patient to be eligible for the study (Textbox 1). After patients consent to participate in the trial (Multimedia Appendix 2), the investigator will determine whether the patient meets the eligibility criteria, and the results of the screening process will be compiled at the investigational site. No eligibility exceptions will be considered. All patients will be considered regardless of race or gender.

Eligible patients can have 1 to 3 painful bone metastases of the extremities, excluding metastases involving the feet, hands, and wrists. Other bone metastases may be treated with conventional radiotherapy while the patient is participating in the study. Patients who have more than 3 painful bone metastases of the limb bones that require treatment are more likely to have generalized pain, which could confound the measurement of pain relief (ie, the response to treatment).

Patients may continue to take steroids during their participation in the clinical trial, if prescribed by their physician. Steroid medication is optional and at the discretion of the prescribing physician.

Because proton range and dosimetry are less certain in the presence of metal, patients with bone fractures or metal implants in the treatment field will be excluded from this study. Patients who will receive cytotoxic chemotherapy within 1 week prior to or 1 week following their planned radiation treatment will also be excluded because concurrent cytotoxic chemotherapy could affect the tissue response to radiation.

Patients interested in participating in the study will be asked to read, understand, and sign the informed consent document, consistent with institutional practices. Patients who meet all eligibility criteria and sign the consent document may proceed onto the study.

If the subject leaves the study for any reason before the next scheduled follow-up visit is completed, the investigator will document the reason(s). In addition, the investigator will attempt to record the overall score of patient-reported pain and scores for pain specifically in treated site(s), use of pain medications, and adverse events (AEs).

**Eligibility criteria.**

**Inclusion criteria**

- Patient age ≥18 years
- Life expectancy of >2 months (per the judgement of the investigator)
- ≤3 painful bone metastases that are in the extremities but are not in the feet, hands, or wrists
- Bone metastases that can be treated using predefined treatment field sizes (7.5 cm × 7.5 cm, 7.5 cm × 10 cm, 7.5 cm × 12 cm, 7.5 cm × 14 cm, 7.5 cm × 16 cm, 7.5 cm × 18 cm, and 7.5 cm × 20 cm), without overlap of radiation fields
- Patients who are able to comply with the protocol
- Provision of signed and dated informed consent form

**Exclusion criteria**

- Patients who are pregnant or nursing
- Prior radiotherapy to the treatment site(s)
- Patients whose painful bone metastasis sites requiring treatment are all in ineligible treatment sites for FLASH, such as lesions of the feet, hands, or wrists or lesions that are not in the extremities
- More than 3 painful bone metastases of the limbs requiring palliative radiotherapy
- Tumor lysis of >50% of the circumferential bone cortex or other factors considered to place the subject at significant risk of pathologic fracture
- Patients with bone fractures
- Patients with metal implants in the treatment field
- Patients who will receive cytotoxic chemotherapy within 1 week prior to or 1 week following their planned radiation treatment
- Prior local therapy modality to the treatment site(s) within 2 weeks of study enrollment
- Patients with pacemakers or other implanted devices at risk of malfunction during radiotherapy
- Patients at known risk of enhanced normal tissue sensitivity to radiotherapy due to inherited predisposition or documented comorbidity that might lead to hypersensitivity to ionizing radiation
- Patients with any other medical condition or laboratory value that would, at the discretion of the investigator, preclude the patient from participation in this clinical investigation
- Patients enrolled in any other clinical studies that the investigator believes to be in conflict with this clinical investigation

An investigator may withdraw a subject’s participation in the study because of the following reasons: patients meeting an exclusion criterion (either newly developed or not previously recognized) that precludes further study participation; significant noncompliance with the study procedure; and the occurrence of an AE, a laboratory abnormality, or any other medical condition or situation such that continued participation in the study would not be in the best interest of the subject.

Subjects who sign the informed consent form and subsequently withdraw or are withdrawn from the study prior to receiving FLASH therapy will not be counted toward the study participant limit and will be replaced. Subjects who sign the informed consent form, receive FLASH therapy, and subsequently withdraw or are withdrawn from the study will be counted toward the study participant limit and will not be replaced.

### Imaging and Treatment Plan Selection

Each subject will undergo computed tomography (CT) simulation imaging for the area(s) encompassing the treatment targets. Prior to the acquisition of the CT simulation image, the subject will be fitted with an immobilization device, such as a Vac-Lok bag (MED-TEC Inc); this will be used at simulation and for reproducing the subject’s positioning at the time of treatment.

The CT simulation images will be transferred to the Eclipse treatment planning workstation (Varian). The target site(s) will be delineated on the transferred images by one of the radiation oncologist investigators. An expansion (margin) of ≥5 mm will be added to the target to create a planning target volume (PTV).

For each of the FLASH treatment sites, plans will be chosen from a predefined library of single-field, 250-MeV transmission beam plans for different field sizes. The largest treatment field available for this study is 7.5 cm × 20 cm. If it is determined at the time of CT simulation or during treatment planning that the target would be inadequately encompassed by the available fields, then the subject no longer meets the eligibility criteria for the study (Textbox 1) and should be removed from the study and replaced.

The predefined plans are designed to deliver a radiation prescription of 8 Gy in a single fraction at a dose rate of ≥40 Gy per second to the PTV. The volume of the PTV receiving 90% of the prescribed dose shall be ≥90%, and the dose to 10% of the PTV will not exceed 110% of the prescription dose. Because a transmission beam is being used, a relative biological effectiveness of 1.0 will be used for the dose calculation, as there is no Bragg peak within the body.

### FLASH Treatment

The treatment plan will be transferred from the ARIA Oncology Information System (Varian) to the proton therapy system console for patient-specific quality assurance (QA) and FLASH radiotherapy delivery. Treatment will be carried out on a ProBeam proton therapy system (Varian). This FDA-cleared proton therapy system will be modified under an IDE to deliver the transmission-beam proton radiation at a FLASH dose rate.

Clinical site staff with appropriate qualifications will be trained on device usage and clinical study operations. The FLASH delivery will be performed in accordance with written instructions and training. QA procedures will be completed to verify that the dose delivered is as prescribed. On each day of treatment, but prior to the subjects’ treatments, QA procedures for the radiotherapy system (machine QA) will be performed to confirm the FLASH dose and the dose rate constancy of the proton delivery system [28]. Additionally, patient-specific QA will be performed per institutional practices for all subjects, using, for example, standard film and ion chamber dosimetry procedures.

At the time of treatment, subjects will be positioned on the treatment couch as planned at the time of their simulation visit. Image guidance will be used to verify that the target is in the correct position for treatment.

### Assessments

#### Overview of Assessments

The following assessments will be conducted over the course of the study and according to the schedule of activities (Table 1). The choice of follow-up visit time points was informed by the study designs of Chow et al [29] and Hartsell et al [9]. Questionnaires will be completed as permitted by the subjects’ clinical status.

**Table 1:** Schedule of assessments during the study.

Steps	Enrollment and baseline^a^	FLASH treatment (day 1)^b^			Post-FLASH treatment follow-up			Follow-up
	
		Before FLASH	FLASH	After FLASH	Day 2^c^	Days 2-11^c^	Each visit^d^	
Patient screening	✓							
Informed consent	✓							
Computed tomography simulation	✓							
Eligibility determination	✓							
Subject and tumor characteristics	✓							
Subject evaluation	✓	✓					✓	
Pain flare questionnaire		✓			✓	✓		
Pain response questionnaire		✓					✓	
FLASH workflow			✓					
Adverse events				✓	✓		✓	

^a^Approximately 1 to 2 weeks before computed tomography simulation.

^b^≤7 days after computed tomography simulation.

^c^The assessments associated with these visits will be completed remotely.

^d^After FLASH treatment (day 1), follow-up visits will occur on day 15 (±2 business days), month 1 (±5 business days), month 2 (±10 business days), month 3 (±10 business days), and every 2 months (±10 business days) thereafter until subject death or loss to follow-up.

#### FLASH Assessment

The following will be documented for each treatment: time that the subject is on the treatment table, any delays in study treatment related to the investigational device (excluding delays due to subject or facility factors not related to study treatment), and any device deficiency.

For each subject, workflow feasibility will be judged as not successful if the total treatment time, measured as subjects’ time on the treatment table, is >1 hour or there is a delay related to the investigational device (excluding delays due to subject or facility factors that are not related to the study treatment) of more than 7 business days from simulation to study treatment.

For this study, a *device deficiency* is defined as an inadequacy of a medical device with respect to its identity, quality, durability, reliability, safety, or performance. Device deficiencies include malfunctions, use errors, and inadequate labeling [30].

In the event of interruption to treatment during FLASH, the remaining portion of the treatment should resume, as the system will correctly execute the remainder of the plan such that the full dose of 8 Gy will be delivered to the target volume. Based on reliability testing, treatment interruption is unlikely to occur.

#### Baseline Characteristics

Baseline characteristics will be assessed before the subjects’ simulation CT and include subject and tumor characteristics.

The subjects’ characteristics are age, gender, performance status, history of medical comorbidities or autoimmune disorders, diagnosis date, and prior cancer-directed treatments.

The tumor characteristics are histology, anatomic location of the original primary tumor, anatomic location of the treatment site(s), target lesion size, target lesion extent of bone circumferential involvement (if available), and metastasis type (ie, lytic, blastic, or mixed).

#### Pain Flare

Transient flare in bone pain at a site treated by radiotherapy is a known acute toxicity of palliative radiotherapy for painful bone metastases in some patients [31,32].

Per the methodology of Chow et al [29], flares in bone pain due to radiation will be assessed by using the Pain Flare Questionnaire (Multimedia Appendix 3), which will be administered on day 1 to day 11. *Pain flare* is defined as either of the following: a minimum of a 2-point increase in the worst pain score for the treated site without a reduction in analgesic intake or a ≥25% increase in analgesic intake based on daily oral morphine equivalence without a reduction in the worst pain score. If the worst pain score before treatment was 9 or 10, the criteria for pain flare are met if the follow-up worst pain score is 10 and reported as worse than the worst pain before treatment with no decrease in analgesic intake. To distinguish pain flare from progression of pain, the worst pain score and analgesic intake have to return to baseline levels during the 11-day (day 1 to day 11) period. The incidence of pain flare will be determined both as the percentage of patients and as the percentage of metastatic treatment sites. Consistent with the Chow et al [29] study, we will document the use of steroid medication.

Data on use of pain medication will be collected, and changes in pain medication use will be evaluated at baseline, during each of the first 10 days after treatment, and during follow-up visits (day 15, month 1, month 2, month 3, and long-term). The percentage of subjects requiring narcotics, nonnarcotic analgesics, and no pain medications will be assessed and compared to literature values from prior clinical trials.

#### Pain Response

Patient-reported pain scores (overall) at baseline and posttreatment (day 15, month 1, month 2, month 3, and long-term) will be assessed, using the Brief Pain Inventory (BPI) Short Form questionnaire (Multimedia Appendix 4). Patient-reported pain scores for each treated site will be assessed using the Treated Sites Pain questionnaire (Multimedia Appendix 5).

The BPI Short Form questionnaire was selected to permit the results of this study to be compared with prior published results that were obtained by using this instrument [9]. Per the methodology of Hartsell et al [9], the worst pain scores in the BPI Short Form and the Treated Sites Pain questionnaire will be used to assess treatment response.

A *complete response* will be defined as having no pain at 3 months after radiation therapy, a *partial response* will be defined as a pain score that is at least 2 points lower than the initial response, a *stable response* will be defined as a 1-point change in pain score in either direction, and a *progressive response* will be defined as a pain score that is at least 2 points higher than the initial score [9].

The percentage of patients and the percentage of metastatic treatment sites having complete, stable, and progressive responses will be determined.

#### Subject Evaluations

Study personnel will review pain medications (including steroid medications), performance status, and findings of physical examinations involving the skin or other normal tissues at the planned treatment site(s) during subject evaluations. The physical evaluation will include photographs of skin at the entry and exit sites of the beam. These should include an image that encompasses the entire anatomic region treated (upper leg, lower leg, upper arm, and lower arm), with close-up photographs of the skin at each treated area(s) if the treated area(s) can be readily identified.

The investigator or a delegate who is a study physician, nurse, physician assistant, or advanced registered nurse practitioner will evaluate and manage AEs. All AEs, regardless of severity or attribution, from the time of treatment and throughout the subjects’ participation in the trial will be captured in the study data. It is expected that most AEs in the study population will be due to underlying cancer and non-FLASH treatments, which will be managed by nonstudy personnel. The investigator (or the person to whom the task is delegated by the investigator) will grade each AE and determine whether the AE is serious per the Common Terminology Criteria for Adverse Events (CTCAE) version 5.0. The investigator will document whether the AE is attributable to FLASH radiotherapy delivery.

The anticipated AEs for this study are the previously documented AEs [9] associated with the treatment of bone metastases via 8 Gy of radiation in 1 fraction at a standard dose rate. It is expected that most frequently seen study-related toxicities will be associated with the skin. Radiation dermatitis will be managed at the study site through outpatient treatment by the investigators or their designees, using an appropriate combination of topical emollients, topical steroids, topical antibiotics, and dressings as deemed necessary by the investigator or designee. For other AEs that might occur, such as the pathologic fracture of the treated bone (a known complication of the disease process), patients will be referred to the appropriate specialist for management.

It is desirable to have in-person follow-up visits. However, in the event of a subject’s inability or reluctance to travel (given the COVID-19 pandemic), it is acceptable to carry out remote follow-up visits. In these circumstances, subject evaluation may be carried out by using remote visits, record reviews, or a combination thereof. Photographs of the treatment site may be taken at home by caregivers, and physical evaluations will be carried out to the extent that is feasible via telehealth.

Toxicities that are rated by the investigator as possibly, probably, or definitely related to FLASH radiotherapy will be used for assessing the toxicity of the FLASH treatment. A *dose-limiting toxicity* (DLT) is defined as a toxicity grade of ³3 that is possibly, probably, or definitely related to FLASH radiotherapy. The number of DLTs will be monitored throughout the duration of the study.

### End of Study

At the end of a subject’s participation in the study, the investigator will document the reason for the end of the study and will attempt to record patient-reported scores for overall pain and for the pain specifically in the treated site(s), the use of pain medications (including steroid medications), and AEs (including skin and other normal tissue toxicities).

### Statistical Analysis

Owing to the small planned sample size, no formal statistical calculations will be performed. The sample size of 10 subjects was determined in consultation with regulatory authorities. Where appropriate, descriptive statistics will be used to analyze the results.

### Privacy and Confidentiality

All efforts will be made to remove patient-identifying information and deidentify the data. Only the minimum necessary information regarding the patients’ health records and treatment while participating in this study will be collected. All reasonable efforts will be made to protect the privacy of the patients.

### Data Collection, Management, and Monitoring

Standardized electronic case report forms and case report form completion guidelines will be created for the collection of study data. These case report forms will include fields for documenting unintended effects of trial interventions or trial conduct, such as AEs, device issues, and protocol deviations.

The study data will be entered into a commercial electronic data capture system designed for clinical research. Automated edit checks, queries, and audit trails are built into the system to ensure accurate data collection. A secure, central storage site provided by the sponsor will be used to collect study DICOM data and photographs.

Data will be transmitted from the study site to the electronic data capture system via a secure internet connection and by using industry-standard encryption modalities. Data access will be password protected.

All data will be reviewed by a clinical monitor from the sponsor or a representative of the sponsor to ensure acceptable accuracy and completeness. The principal investigator will maintain adequate and accurate records to enable the conduct of the study to be fully documented and the study data to be subsequently verified. After study closure, the investigator will maintain all source documents and study-related documents.

### Stopping Rules

Enrollment will cease and the study data will be reviewed by the study committee (principal investigator and coinvestigators) and by the Cincinnati Cancer and Blood Disease Institute Data Safety Monitoring Board (DSMB) if any of the following occurs: 3 subjects experience a DLT, 3 subjects are on the treatment table for more than 1 hour, 3 subjects have a delay in study treatment related to the investigational device (excluding delays due to subject or facility factors not related to the study treatment) of more than 7 business days from simulation to treatment, or a major device malfunction in dose delivery (as indicated by the dose monitoring system) occurs. The DSMB is independent of the sponsor.

## Results

FAST-01 opened to enrollment on November 3, 2020, and the final patient signed the informed consent document on October 1, 2021.

Data collection will continue for the lifetime of the study participants or when they withdraw from follow-up. Interim results, including the conclusions of the workflow feasibility analysis, are expected to be published in 2022. The final study results will be published upon completion of the study, which may run from 1.5 years to several years, depending on the duration of subject participation.

## Discussion

This first-in-human study investigates the use of ultrahigh dose rate proton FLASH therapy for the single-fraction treatment of painful bone metastases in the extremities in a routine clinical setting. Numerous preclinical studies have shown that FLASH therapy reduces radiation-related toxicities when compared to conventional radiotherapy. In this trial, we anticipate that the feasibility of FLASH therapy in a clinical setting will be demonstrated. Additionally, we hypothesize that radiation-induced toxicity will be limited and that pain relief will be similar to historical comparators.

The sample size of 10 subjects in this clinical trial is common in first-in-human device studies, and it was the sample size agreed upon with the US FDA as part of an IDE approval.

This study will use single-fraction treatment delivered at a dose rate of ≥40 Gy per second for the enrolled subjects. Single-fraction radiotherapy for the treatment of bone metastases has been shown to be effective in prior investigations. In 2005, a prospective, phase 3, randomized RTOG study compared the efficacy of multiple-fraction radiotherapy (300 cGy/fraction × 10 fractions) with single-fraction radiotherapy (800 cGy/fraction × 1 fraction) for the treatment of painful bone metastases. The results indicated comparable efficacy for the two fractionation schedules [9]. Based on ongoing reviews of the clinical evidence [4,9,33], single-fraction, 8-Gy radiotherapy remains a standard of care for the palliation of bone metastasis, and it was therefore selected as an appropriate course of treatment for an initial clinical trial of FLASH. The use of this fractionation for the FLASH treatment enables comparison with the data from prior investigations that used photon radiation and conventional dose rates.

AEs attributed to FLASH will be compared with those observed in the prior RTOG randomized trial [9]. In that study, toxicities were scored by using the RTOG acute and late morbidity criteria. That grading system has since been supplanted by the CTCAE system. For skin toxicities, which are expected to be the most common treatment-related toxicity in our clinical study, the RTOG skin and CTCAE radiation dermatitis scoring systems are very similar, and direct toxicity comparisons can be made between our study and historical studies.

The BPI questionnaire (both the long and the short form questionnaires) was originally developed for patients with cancer and consists of a set of standardized questions for describing pain, pain medication use, and the interference of pain in the patients’ lives. The BPI has been recommended as an appropriate tool for measuring pain from metastatic bone disease [34], and using it makes it possible to consider the data from this study in the context of historical data. Similarly, the pain flare questionnaire chosen for use in this evaluation is the same instrument used by Chow et al [29] in a prior study evaluating the use of steroids in combination with radiotherapy for the palliation of bone metastases.

FAST-01 is designed as a first-in-human trial involving 10 patients requiring palliative radiotherapy to the extremities. This low-risk clinical setting is ideal for the initial evaluation of this new technology. Favorable results would support the further investigation of FLASH therapy for other clinical indications. Future research could include treating lesions nearer to sensitive normal tissues and evaluating curative dose regimens.

## Appendix

Multimedia Appendix 1 Trial recruitment flier.

Multimedia Appendix 2 Consent form.

Multimedia Appendix 3 Pain flare assessment.

Multimedia Appendix 4 Overall pain assessment.

Multimedia Appendix 5 Treated Sites Pain questionnaire.

## Abbreviations

AE: adverse event
BPI: Brief Pain Inventory
CT: computed tomography
CTCAE: Common Terminology Criteria for Adverse Events
DLT: dose-limiting toxicity
DSMB: Data Safety Monitoring Board
FDA: Food and Drug Administration
IDE: investigational device exemption
PTV: planning target volume
QA: quality assurance
RTOG: Radiation Therapy Oncology Group

## Data Availability

Data sharing is not applicable to this manuscript, as no data sets were generated or analyzed during the development of the protocol described herein. Once data collection begins, all investigators will have access to anonymized patient-level data.
