# Supplementary material for: FLASH Radiotherapy for the Treatment of Symptomatic Bone Metastases (FAST-01): Protocol for the First Prospective Feasibility Study
Source: JMIR Res Protoc. 2023 Jan 5;12:e41812. doi: 10.2196/41812 (PMC9893728; doi:10.2196/41812)
Supplement: Multimedia Appendix 1 [file resprot_v12i1e41812_app1.pdf]

Study title: Feasibility Study of FLASH Radiotherapy for the Treatment of Symptomatic Bone Metastases (FAST-01)

Principal Investigator: Dr. John Breneman
Study sponsor: Varian Medical Systems

Brief study summary: FLASH is a new, experimental way to give proton radiation therapy. Research in animals indicate fewer side effects with FLASH compared to the regular way we currently deliver proton radiation treatments. The purpose of this study is to assess the efficacy of FLASH radiation therapy for patients with painful bone metastases in their extremities, as well as other safety factors.

Key criteria for study participation:

Inclusion Criteria:

- Age at least 18 years
- Up to 3 painful bone metastasis(-es) in the extremities

Exclusion Criteria:

- Prior radiotherapy to the same treatment site(s)
- Bone metastases of the feet, hands, wrists are not currently eligible for treatment on this FLASH trial
- Bone fractures and/or metal implants in the treatment field
- Chemotherapy given within 1 week prior to or 1 week following the planned radiation treatment
- Presence of pacemakers or other implanted devices (defibrillators) =

Additional study information:
You may be eligible to receive some financial support to help cover expenses for your time and effort.

For more information about this study or to see if you are eligible to participate in this study, please contact: Dr. John Breneman (John.Breneman@uc.edu), Dr. Emily Daugherty (Emily.Daugherty@uc.edu), or Cincinnati Children's research team (cancer@cchmc.org).
