# Supplementary material for: FLASH Radiotherapy for the Treatment of Symptomatic Bone Metastases (FAST-01): Protocol for the First Prospective Feasibility Study
Source: JMIR Res Protoc. 2023 Jan 5;12:e41812. doi: 10.2196/41812 (PMC9893728; doi:10.2196/41812)
Supplement: Multimedia Appendix 2 [file resprot_v12i1e41812_app2.pdf]

Title of Research Study: Feasibility Study of FLASH Radiotherapy for the Treatment of Symptomatic Bone Metastases (FAST-01) (VAR-2019-02)

Key Information:

The following is a short summary of this study to help you decide whether to be a participant in it. More detailed information about the study is listed later in this form. This document does not replace the discussion you should have with the research team about this study including having any questions or concerns answered.

This is a consent form. It explains this research study. If you decide that you want to be in this research study, then you will sign this form to show that you agree to be part of this study. If you sign this form, you will receive a signed copy of this form for your records.

Principal Investigator:
John Breneman, MD

Contact Info:
(513) 517-CBDI (2234)

Industry Protocol #:
VAR-2019-02

Drug Name:
N/A

Funding:
Varian Medical Systems

Reason for the study:

A goal of this study is to evaluate if a new way to deliver radiation therapy, called FLASH proton therapy, can efficiently provide pain relief for patients with bone metastases. Another goal is to measure the side effects from this new way of delivering radiation therapy. Proton therapy is a type of radiation therapy that uses protons (positively charged particles) to treat tumors. It is just as effective as traditional x-ray beam radiation therapy, but since it uses a different way of making radiation it can be delivered at faster dose rates (same amount of radiation over a shorter period of time) than traditional radiation therapy. FLASH radiotherapy is the term used for this form of faster dose rate radiation therapy.

FLASH radiotherapy has been used in treatment of tumors in animals and early results have demonstrated fewer side effects than traditional radiotherapy. However, FLASH radiotherapy using protons has not been evaluated in humans prior to this research study. To date, FLASH radiotherapy has been administered to one human patient with lymphoma involving the skin using a different form of FLASH radiotherapy involving electrons instead of protons. This one patient had a rapid and complete response of the lymphoma with mild and temporary skin irritation using this form of FLASH radiotherapy. This patient was treated for a different condition than bone metastasis. Therefore, this patient's results may not apply to you.

This research study will evaluate whether FLASH radiotherapy can provide pain relief for patients with bone metastasis. You are being asked to take part in this study because you have a painful bone metastasis in the arm and/or leg. The FLASH radiotherapy will be delivered to the painful bone metastasis. The FLASH radiotherapy is not intended as

treatment for your original tumor. Traditional radiation treatment may still be used for pain relief of other metastases.

FLASH radiotherapy is considered experimental because it has not been approved by the Food and Drug Administration (FDA).

Varian Medical Systems is the sponsor of this study.

Procedures:
If you are eligible to participate in this study, and if you agree to participate, you will receive one treatment with FLASH radiotherapy. This treatment will take place at the Cincinnati Children's Proton Therapy Center (7777 Yankee Road, Liberty Township, OH 45044).

More detailed information about the study procedures can be found under ***"(Detailed Procedures)"***.

Risks to Participate:
The types of side effects of FLASH radiotherapy are expected to be similar to those resulting from traditional radiation treatment at standard dose rates. Such side effects could include:

- Potential skin changes in the treatment area
    - Red skin
    - Weeping of skin
    - Skin ulcers
    - Breakdown of the skin and tissues underneath the skin
    - Thickening or scarring of the skin
    - Changes in skin color (skin color becomes darker or lighter)
    - Hair loss in the treated area
- Potential swelling in the treated arm and/or leg (lymphedema)
- Potential damage to other tissues in the treatment area, including to muscle, nerve, and/or bone
- Fracture at the metastasis site
- Decreased blood cell count
- Second cancer (such as leukemia) that is different from the kind of cancer you have now

Another potential risk would be inadequate pain relief.

Participation in this study may or may not affect your ability to participate in other research treatment options.

Benefits to Participate:
We cannot promise any benefits to you from your taking part in this research. However, experimental results in animal studies suggest that FLASH radiotherapy may result in less harm to normal tissues compared to traditional radiation therapy.

Other Options:
Participation in research is completely voluntary. Your decision to participate or not to participate will not affect the care you receive.

If you choose not to participate, you will receive traditional radiation treatment. Your doctor will discuss with you traditional methods for radiation treatment.

Cost to Participate:
Some of the tests or treatments used in this study may be part of standard care used to maintain your health even if you do not take part in this study. You or your insurance company will be responsible for the cost of this standard care.

The sponsor will pay for your FLASH radiotherapy.

Payment:
If you agree to take part in this research study, you will be paid up to $400 for your time and effort for completion of this study. You will receive $200 after you complete your routine CT scan of the involved area, and $200 after you complete the Month 3 visit. If you do not finish the study, you will be paid only for the portion of the study you have completed. This compensation will be loaded onto a debit card called ClinCard and you will receive a handout that explains how to use it.

Because you are being paid for your participation, Cincinnati Children's is required by the Internal Revenue Service (IRS) to collect and use your social security number (SSN) or taxpayer identification number (TIN) to track the amount of money that we pay. You will need to complete a Federal W-9 form for this income tax reporting. This form requires your Social Security number. This form will be given to the Cincinnati Children's business office. It will not be kept as part of your study chart. If you move, you will need to complete another W-9 with an updated address.

If you live more than 50 miles away from Cincinnati Children's, you can be reimbursed for costs associated with your travel. We will reimburse up to $150 per day (for a maximum of 3 days) for mileage, food and lodging costs. Please save your receipts and give them to your care manager for reimbursement.

If you prefer, you may choose to participate in the study and choose not to receive any payment.

Additional Study Information:
The following is more detailed information about this study in addition to the Key Information.

If I have Questions or would like to know about:

**If I have questions or would like to know about …**	**You can call …**	**At …**
• Emergencies<br>• General treatment questions<br>• Research-related injuries<br>• Any research concerns or complaints	**Cancer and Blood Diseases Institute Triage Operator**	Phone: (513) 517-CBDI (2234)<br>Ask for the oncology physician on call
• Your rights as a participant	**Institutional Review Board**<br><br>This is a group of scientists and community members who make sure research meet legal and ethical standards.	Phone: (513) 636-8039

A description of this clinical trial is available on http://www.ClinicalTrials.gov, as required by U.S. Law. This website will not include information that can identify you. At most, the website will include a summary of the results. You can search this website at any time.

You will get a copy of this consent form. If you want more information about this study, ask your study doctor.

Total number of participants:

About 10 study participants will be taking part in this study at Cincinnati Children's Hospital Medical Center.

Detailed Procedures:

If your doctor determines that your bone metastasis(-es) are likely to be treatable using FLASH radiotherapy, you will have a routine CT scan done of the involved area(s). This scan is used for planning radiation treatment, and is necessary regardless of your participation in the study. Your doctor will use the results of the CT scan to decide if your bone metastasis(-es) are suitable for treatment using FLASH radiotherapy in this study.

There are limitations to how large an area or how deep an area in your body can be treated using FLASH radiotherapy in this study. If the measurements on the CT scan show that your tumor or body dimensions are too large, then FLASH treatment would not be an option and you would not continue on this study.

Female participants of childbearing age will be given a urine pregnancy test prior to the CT scan.

If you are eligible and choose to participate, FLASH treatment will take place within 7 business days after your CT scan. On the day of treatment, but before you receive treatment, you will complete questionnaires reporting your pain overall, pain at the planned treatment site(s), and pain and steroid medication usage. You will have a physical examination of the planned treatment sites, including photographs of the planned treatment sites and where the treatment sites are located (upper leg, lower leg, upper arm, lower arm). You will then receive the FLASH radiotherapy for this study. The treatment session should take less than one hour.

Up to 3 bone metastases sites can be treated on this protocol in one treatment visit.

Once your radiation treatment is complete, for the first 10 days after treatment you will complete a questionnaire daily reporting your pain at the treated site(s) and pain medication usage.

On the day after treatment, you will have a remote visit to check for any side effects from the treatment.

After your treatment, you will have follow-up visits at 2 weeks, 1 month, 2 months, 3 months, then every 2 months after the 3-month visit indefinitely. These visits may be in person or may be done remotely depending on your ability and/or willingness to travel.

At these visits: you will complete questionnaires reporting your pain overall and at the treated site(s); you will be checked for any side effects from the treatment; your performance status will be checked; the pain and steroid medication you are using will be recorded. For the in-person visits, you will have a physical examination, including photographs of the treated sites and where the treated sites are located (upper leg, lower leg, upper arm, lower arm). Some of these visits may be part of your routine radiation treatment follow-up visits.

We would like to continue collecting the follow-up information on you for the rest of your life.

Detailed Risks

The side effects listed above are based on side effects reported in patients who received traditional x-ray beam radiation treatment. If you notice any of these side effects notify your study nurse or doctor right away.

If the FLASH radiotherapy is interrupted (as sometimes can occur with traditional radiation treatment at standard dose rates), the treatment can be resumed to complete delivery of the original prescribed dose. The dose rate for the remainder of the treatment fraction may be lower than the dose rate for the portion of the treatment fraction prior to the interruption. The potential normal tissue sparing benefit of the FLASH dose rate may

be less. However, treatment can be completed likely without any reduced effect in treating the tumor. Based on the reliability history of our proton machine, treatment interruption is unlikely to occur.

You will have regular follow-up visits to check for any side effects. If you experience a side effect, the study staff may give you medicines to help lessen these effects. Side effects may be mild, moderate, or serious. Some side effects go away after treatment and some side effects may persist.

In some cases, side effects can be serious, long-lasting, may never go away, and may lead to death. Cases of sudden death have occurred in patients with advanced cancer.

There may be other side effects that may happen that are not known.

<u>Possible Risk to an Unborn Child</u>
If you are pregnant or nursing, you cannot be in this research study because radiation may harm your baby. Patients who agree to participate in this study should not become pregnant while on this study. Patients and their sexual partners should avoid sex and/or use two effective methods of contraception that is medically appropriate based on your personal doctor's recommendation at that time and should be used through the time you receive FLASH treatment. If you or your partner becomes pregnant through the time you receive FLASH treatment, please notify your doctor immediately. For more information about risks and side effects, ask your doctor.

Change of Mind/Study Withdrawal:

Your participation in this study is voluntary. You may decide not to participate or you may leave the study at any time. You can do this by telling the study doctor or sending written notice. Your decision will not be held against you or result in any penalty or loss of benefits to which you are entitled.

If you stop being in the research, data already collected may not be removed from the study database. You will be asked whether the investigator can collect data from your routine medical care. If you agree, this data will be handled the same as research data.

Your participation in this study may be stopped at any time by the study doctor or the Sponsor without your approval for any of the following reasons:

- if it is in your best interest;
- you do not consent to continue in the study after being told of changes in the research that may affect you;
- or for any other reason.

The Sponsor may stop or suspend the study at any time.

We will tell you about any new information that may affect your health, welfare, or choice to stay in the research. You may be asked to sign a new consent form if this occurs.

Privacy:

Efforts will be made to limit the use and disclosure of your personal information, including research study and medical records, to people who have a need to review this information. We cannot promise complete privacy. Organizations that may inspect and copy your information include the IRB and other representatives of this organization, the Sponsor, and U.S. Food and Drug Administration (FDA). "Sponsor" means any persons or companies that are working for or with the Sponsor, or owned by the Sponsor.

The Sponsor, monitors, auditors, and the IRB will be granted direct access to your medical and study records to conduct and oversee the research. By signing this document, you are authorizing this access. Also, the FDA, U.S. Department of Health and Human Services (DHHS) and other agencies within DHHS, governmental agencies to whom certain diseases (reportable diseases) must be reported, and governmental agencies in other countries may be given access to your records for research or regulatory purposes.

The results of this research may be published or presented at meetings. Data collected for or generated from this study could be shared and used for future research. Data may be shared with other collaborators at Cincinnati Children's and possibly with outside collaborators, who may be at another institution or for-profit company. The Sponsor may use the study data and information for training materials, promotional materials, submissions to regulatory authorities, and in discussions with scientific advisors and other clinicians working with the Sponsor. The Sponsor may also use the data:

- To study and improve the performance of the FLASH radiotherapy
- To assure the safety, effectiveness, and quality of research and of medical products and therapies, which may include, but is not limited to, the collection and reporting of adverse event information as permitted by law
- To conduct new medical research and develop proposals for new medical proposals for new medical products or therapies
- As required by law

All the study data may be used beyond the duration of the study.

Your name and other identifying information will be kept confidential. Total confidentiality cannot be guaranteed because of the need to give information to the parties described above.

If injured while in the study:

If you believe that you have been injured as a result of this research, you should contact (513) 517-CBDI (2234) and ask for the oncology physician on call as soon as possible to discuss the concerns. Treatment for injuries is available at CCHMC.

If you go to the Emergency Room or to another hospital or doctor it is important that you tell them that you are in a research study. If possible, you should give them a copy of this consent form.

Cincinnati Children's Hospital follows a policy of making all decisions about compensation for the medical treatment of physical injuries that happened during or were caused by research on an individual basis.

AUTHORIZATION FOR USE/DISCLOSURE OF HEALTH INFORMATION FOR RESEARCH

To be in this research study you must also give your permission (or authorization) to use and disclose (or share) your "protected health information" (called PHI for short).

What protected health information will be used and shared during this study?

Cincinnati Children's Hospital Medical Center (Cincinnati Children's) will need to use and share your PHI as part of this study. This PHI will come from:

- Your Cincinnati Children's medical records
- Your research records

The types of information that will be used and shared from these records include:

- Laboratory test results, diagnosis, and medications
- Reports and notes from clinical and research observations
- Imaging (like CT scans, MRI scans, x-rays, etc.) studies and reports
- If applicable, information concerning HIV testing or the treatment of AIDS or AIDS-related conditions

Who will share, receive and/or use your protected health information in this study?

- Staff at all the research study sites (including Cincinnati Children's)
- Personnel who provide services to you as part of this study
- Other individuals and organizations that need to use your PHI in connection with the research, including people at the sponsor and organizations that the Sponsor may use to oversee or conduct the study.
- The members of the Cincinnati Children's Institutional Review Board and staff of the Office of Research Compliance and Regulatory Affairs.

How will you know that your PHI is not misused?

People that receive your PHI as part of the research are generally limited in how they can use your PHI. In addition, most people who receive your PHI are also required by federal privacy laws to protect your PHI. However, some people that may receive your PHI may not be required to protect it and may share the information with others without your permission, if permitted by the laws that apply to them.

Can you change your mind?

You may choose to withdraw your permission at any time. A withdrawal of your permission to use and share your PHI would also include a withdrawal from participation in the research study. If you wish to withdraw your permission to use and share PHI you need to notify the study doctor, listed on the first page of this document, in writing. Your request will be effective immediately and no new PHI about you will be used or shared.

The only exceptions are (1) any use or sharing of PHI that has already occurred or was in process prior to you withdrawing your permission and (2) any use or sharing that is needed to maintain the integrity of the research.

Will this permission expire?

Your permission only applies to PHI received, used and disclosed during this study. This permission does not expire once your participation in the study ends. We can still use this PHI after the study ends in the ways described in this document, including for future research purposes. Your permission will remain valid until and unless it is revoked by you according to the procedures described in the "Can you change your mind?" section of this document.

SIGNATURES

The research team has discussed this study with you and answered all of your questions. Like any research, the researchers cannot predict exactly what will happen. Once you have had enough time to consider whether you should participate in this research, you will document your permission by signature below.

By signing this consent form, you are authorizing the use and disclosure of your information by the Institution, the Sponsor and others as described above.

You will receive a copy of this signed document for your records.

____
Printed Name of Research Participant

____________________________________ ____________
Signature of Research Participant Indicating Consent — Date

____________________________________ ____________
Signature of Parent or Legally Authorized Representative* — Date

____
* If signed by a legally authorized representative, a description of such representative's authority must be provided

____________________________________ ____________
Signature of Individual Obtaining Consent — Date
