# Supplementary material for: FLASH Radiotherapy for the Treatment of Symptomatic Bone Metastases (FAST-01): Protocol for the First Prospective Feasibility Study
Source: JMIR Res Protoc. 2023 Jan 5;12:e41812. doi: 10.2196/41812 (PMC9893728; doi:10.2196/41812)
Supplement: Multimedia Appendix 3 [file resprot_v12i1e41812_app3.pdf]

FRM-07 Pain Flare Questionnaire

1.0 Date and time

Today's date:

MM/dd/yyyy Now

Time when you started this questionnaire:

HH:MM AM Now

2.0 Treated Site #1

Description of treatment site #1

left arm

2.1 For this treated site, please rate your worst pain over the last 24 hours

0 1 2 3 4 5 6 7 8 9 10

No Pain

Pain as bad as you can imagine

2.2 For this treated site, please indicate how your worst pain over the last 24 hours compared to your worst pain in this site on the day of your radiation treatment

- ○ Worse
- ○ Same
- ○ Better

Continue

FRM-07 Pain Flare Questionnaire

3.0 Treated Site #2 (if applicable)

Description of treatment site #2

right arm

3.1 For this treated site, please rate your worst pain over the last 24 hours

0 1 2 3 4 5 6 7 8 9 10

No Pain

Pain as bad as you can imagine

3.2 For this treated site, please indicate how your worst pain over the last 24 hours compared to your worst pain in this site on the day of your radiation treatment

- ○ Worse
- ○ Same
- ○ Better

Back Continue

FRM-07 Pain Flare Questionnaire

4. Treated Site #3 (if applicable)

Description of treatment site #3

N/A

4.1 For this treated site, please rate your worst pain over the last 24 hours

0 1 2 3 4 5 6 7 8 9 10

No Pain

Pain as bad as you can imagine

4.2 For this treated site, please indicate how your worst pain over the last 24 hours compared to your worst pain in this site on the day of your radiation treatment

- ○ Worse
- ○ Same
- ○ Better

Back Continue
