# Supplementary material for: FLASH Radiotherapy for the Treatment of Symptomatic Bone Metastases (FAST-01): Protocol for the First Prospective Feasibility Study
Source: JMIR Res Protoc. 2023 Jan 5;12:e41812. doi: 10.2196/41812 (PMC9893728; doi:10.2196/41812)
Supplement: Multimedia Appendix 4 [file resprot_v12i1e41812_app4.pdf]

FRM-08 Brief Pain Inventory Short Form

1.0 Date and time

Today's date:

MM/dd/yyyy Now

Time when you started this questionnaire:

HH:MM AM Now

1.1 Throughout our lives, most of us have had pain from time to time (such as minor headaches, sprains, and toothaches). Have you had pain other than these everyday kinds of pain today?

- ○ Yes
- ○ No

Continue

FRM-08 Brief Pain Inventory Short Form

1.2 Referring to the diagram, indicate the areas where you feel pain by selecting the number checkbox for each area. Indicate the one area that hurts the most by selecting the number for that area.

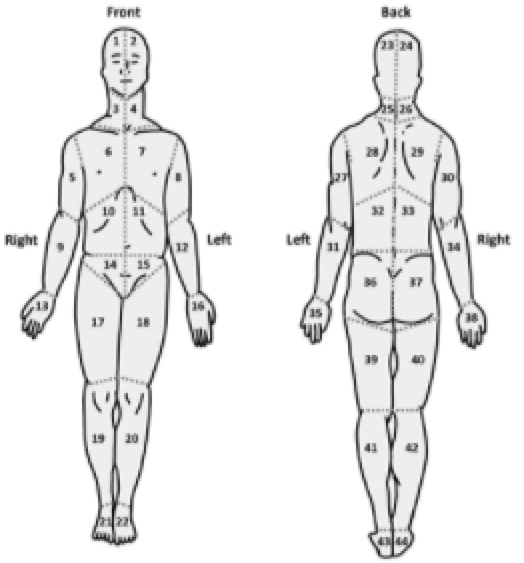

Site #s - Front	Site #s - Back
☐ 1	☐ 23
☐ 2	☐ 24
☐ 3	☐ 25
☐ 4	☐ 26
☐ 5	☐ 27
☐ 6	☐ 28
☐ 7	☐ 29
☐ 8	☐ 30
☐ 9	☐ 31
☐ 10	☐ 32
☐ 11	☐ 33
☐ 12	☐ 34
☐ 13	☐ 35
☐ 14	☐ 36
☐ 15	☐ 37
☐ 16	☐ 38
☐ 17	☐ 39
☐ 18	☐ 40
☐ 19	☐ 41
☐ 20	☐ 42
☐ 21	☐ 43
☐ 22	☐ 44
☐ N/A	☐ N/A

Back Continue

FRM-08 Brief Pain Inventory Short Form

What area hurts the most?

Site #:

- ○ 1
- ○ 2
- ○ 3
- ○ 4
- ○ 5
- ○ 6
- ○ 7
- ○ 8
- ○ 9
- ○ 10
- ○ 11
- ○ 12
- ○ 13
- ○ 14
- ○ 15
- ○ 16
- ○ 17
- ○ 18
- ○ 19
- ○ 20
- ○ 21
- ○ 22
- ○ 23
- ○ 24
- ○ 25
- ○ 26
- ○ 27
- ○ 28
- ○ 29
- ○ 30
- ○ 31
- ○ 32
- ○ 33
- ○ 34
- ○ 35
- ○ 36
- ○ 37
- ○ 38
- ○ 39
- ○ 40
- ○ 41
- ○ 42
- ○ 43
- ○ 44

Back Continue

FRM-08 Brief Pain Inventory Short Form

The next four questions, 1.3 – 1.6, relate to your pain in general, not just the area that hurts the most.

1.3 Please rate your pain by selecting the one number that best describes your pain at its worst in the last 24 hours.

0 1 2 3 4 5 6 7 8 9 10

No Pain

Pain as bad as you can imagine

1.4 Please rate your pain by selecting the one number that best describes your pain at its least in the last 24 hours.

0 1 2 3 4 5 6 7 8 9 10

No Pain

Pain as bad as you can imagine

Back Continue

FRM-08 Brief Pain Inventory Short Form

1.5 Please rate your pain by selecting the one number that best describes your pain on the average.

0 1 2 3 4 5 6 7 8 9 10

No Pain — Pain as bad as you can imagine

1.6 Please rate your pain by selecting the one number that tells how much pain you have right now.

0 1 2 3 4 5 6 7 8 9 10

No Pain — Pain as bad as you can imagine

Back Continue

FRM-08 Brief Pain Inventory Short Form

1.7 What treatments or medications are you receiving for your pain?

1.8 In the last 24 hours, how much relief have pain treatments or medications provided? Please select the one percentage that most shows how much relief you have received.

0% 10% 20% 30% 40% 50% 60% 70% 80% 90% 100%

No Relief

Complete Relief

Back Continue

FRM-08 Brief Pain Inventory Short Form

1.9 Select the one number that describes how, during the past 24 hours, pain has interfered with your:

A. General Activity

0 1 2 3 4 5 6 7 8 9 10

Does not Interfere — Completely Interferes

B. Mood

0 1 2 3 4 5 6 7 8 9 10

Does not Interfere — Completely Interferes

Back Continue

FRM-08 Brief Pain Inventory Short Form

C. Walking Ability

0 1 2 3 4 5 6 7 8 9 10

Does not Interfere — Completely Interferes

D. Normal Work (includes both work outside the home and housework)

0 1 2 3 4 5 6 7 8 9 10

Does not Interfere — Completely Interferes

Back Continue

FRM-08 Brief Pain Inventory Short Form

E. Relations with other people

0 1 2 3 4 5 6 7 8 9 10

Does not Interfere — Completely Interferes

F. Sleep

0 1 2 3 4 5 6 7 8 9 10

Does not Interfere — Completely Interferes

G. Enjoyment of life

0 1 2 3 4 5 6 7 8 9 10

Does not Interfere — Completely Interferes

Copyright 1991 Charles S. Cleeland, PhD
Pain Research Group
All rights reserved

Back Continue
