# Supplementary material for: FLASH Radiotherapy for the Treatment of Symptomatic Bone Metastases (FAST-01): Protocol for the First Prospective Feasibility Study
Source: JMIR Res Protoc. 2023 Jan 5;12:e41812. doi: 10.2196/41812 (PMC9893728; doi:10.2196/41812)
Supplement: Multimedia Appendix 5 [file resprot_v12i1e41812_app5.pdf]

FRM-09 Treated Sites Pain Questionnaire

1.0 Date and time

Today's date:

MM/dd/yyyy

Time when you started this questionnaire:

HH:MM

2.0 Treated site #1

Description of treatment site #1

2.1 Please rate your pain by selecting the one number that best describes your pain at its worst in the last 24 hours

0 1 2 3 4 5 6 7 8 9 10

No Pain — Pain as bad as you can imagine

2.2 Please rate your pain by selecting the one number that best describes your pain at its least in the last 24 hours

0 1 2 3 4 5 6 7 8 9 10

No Pain — Pain as bad as you can imagine

Continue

FRM-09 Treated Sites Pain Questionnaire

2.3 Please rate your pain by selecting the one number that best describes your pain on the average

0 1 2 3 4 5 6 7 8 9 10

No Pain

Pain as bad as you can imagine

2.4 Please rate your pain by selecting the one number that tells how much pain you have right now

0 1 2 3 4 5 6 7 8 9 10

No Pain

Pain as bad as you can imagine

Back Continue

Varian Confidential

FRM-09 Treated Sites Pain Questionnaire

3. Treated Sites site #2 (if applicable)

Description of treatment site #2

3.1 Please rate your pain by selecting the one number that best describes your pain at its worst in the last 24 hours

0 1 2 3 4 5 6 7 8 9 10

No Pain — Pain as bad as you can imagine

3.2 Please rate your pain by selecting the one number that best describes your pain at its least in the last 24 hours

0 1 2 3 4 5 6 7 8 9 10

No Pain — Pain as bad as you can imagine

Back Continue

FRM-09 Treated Sites Pain Questionnaire

3.3 Please rate your pain by selecting the one number that best describes your pain on the average

0 1 2 3 4 5 6 7 8 9 10

No Pain

Pain as bad as you can imagine

3.4 Please rate your pain by selecting the one number that tells how much pain you have right now

0 1 2 3 4 5 6 7 8 9 10

No Pain

Pain as bad as you can imagine

Back Continue

Varian Confidential

FRM-09 Treated Sites Pain Questionnaire

4. Treated Sites site #3 (if applicable)

Description of treatment site #3

4.1 Please rate your pain by selecting the one number that best describes your pain at its worst in the last 24 hours

0 1 2 3 4 5 6 7 8 9 10

No Pain — Pain as bad as you can imagine

4.2 Please rate your pain by selecting the one number that best describes your pain at its least in the last 24 hours

0 1 2 3 4 5 6 7 8 9 10

No Pain — Pain as bad as you can imagine

Back Continue

FRM-09 Treated Sites Pain Questionnaire

4.3 Please rate your pain by selecting the one number that best describes your pain on the average

0 1 2 3 4 5 6 7 8 9 10

No Pain

Pain as bad as you can imagine

4.4 Please rate your pain by selecting the one number that tells how much pain you have right now

0 1 2 3 4 5 6 7 8 9 10

No Pain

Pain as bad as you can imagine

Back Continue
